# A Joint Approach for Energy Replenishment and Data Collection with Two Distinct Types of Mobile Chargers in WRSN

**DOI:** 10.3390/s25030956

**Published:** 2025-02-05

**Authors:** Yuxiang Li, Tianyi Shao, Weixin Gao, Feng Lin

**Affiliations:** College of Computer Science, Sichuan University, Chengdu 610065, China; liyuxiang1@stu.scu.edu.cn (Y.L.); shaotianyi30@stu.scu.edu.cn (T.S.); gaoweixin1@stu.scu.edu.cn (W.G.)

**Keywords:** wireless rechargeable sensor network, data collection, energy replenishment, mobile charger

## Abstract

Wireless rechargeable sensor networks (WRSNs) address the energy scarcity problem in wireless sensor networks by introducing mobile chargers (MCs) to recharge energy-hungry sensor nodes. Scheduling MCs to charge the recharge nodes is the primary focus of the energy replenishment scheme in WRSNs. The performance of the energy replenishment scheme is significantly influenced by the energy level of each node, which is depends on the data collection scheme employed by the network. Consequently, integrating energy replenishment and data collection has become a new concern in WRSN research. However, the MCs’ workload and travel time increase when data collection and energy replenishment are performed simultaneously, leading to an increase in both the node’s charging delay and data collection delay. In this work, our goal is to reduce the delays in data collection and node charging by proposing a new joint energy replenishment and data collection approach. In the proposed approach, certain nodes in the network are selected as data storage nodes to temporarily store all the collected data based on their geographical locations. A special class of MCs, called MCDs (mobile charger and data collectors), is then assigned the responsibility of charging these data storage nodes and collecting the data stored. Afterwards, the task of recharging the remaining network nodes falls to another type of MC. By combining the capabilities of two distinct MC types, the workload and the travel distance of MCs are reduced. When compared to the conventional joint algorithms, the simulation results demonstrate that the proposed approach successfully decreases the delay it takes to gather data and recharge nodes.

## 1. Introduction

Wireless rechargeable sensor networks (WRSNs) [1] can extend the lifetime of wireless sensor networks by introducing mobile chargers to charge sensor nodes with wireless power transfer technologies [2,3,4,5]. As a promising solution to the energy scarcity problem in wireless sensor networks, WRSNs have garnered significant research attention [6].

The majority of research on energy replenishment approaches in WRSNs has focused on the problem of efficiently scheduling MCs to charge sensor nodes based on their energy levels [7,8,9]. On the other hand, the energy consumption of sensor nodes is primarily driven by inter-node communication [10]. Consequently, the network’s data collection scheme significantly impacts node energy levels as well as, in turn, MCs’ scheduling. Therefore, integrating data collection into the energy replenishment process can enhance MC scheduling efficiency and has emerged as a new research concern in WRSNs.

**Figure 1:**
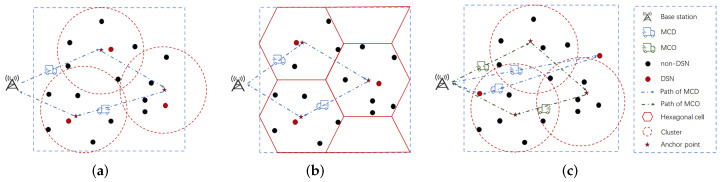
Three energy replenishment and data collection schemes. Where (**a**) depicts the clustering algorithm to group nodes, (**b**) depicts the hexagonal regions to group nodes and (**c**) shows the proposed scheme to group nodes.

Several studies have focused on data collection schemes for wireless sensor networks that utilize mobile assisted nodes. These mobile assisted nodes periodically travel from the base station to the area around the sensor nodes, collect the data sensed by those nodes, and then go back to the base station to finish delivering the data. Naturally, this concept has been used in several WRSN studies to accomplish the joint energy replenishment and data collection by enabling the MCs to retrieve the data sensed by the charged nodes during their charging travelling. As illustrated in Figure 1a,b, most of these existing joint energy replenishment and data collection approaches use clustering algorithms [11,12] or partition the network area into hexagonal cells [13,14] to group nodes according to the MC’s charging radius. The MCs use the geometric centers of these clusters/cells as the anchor points along their traveling paths. As a result, when one MC moves to an anchor point, all nodes in the corresponding cluster/cell fall within its charging range, allowing the MC to charge them. Meanwhile, the MC can also communicate with these nodes at the anchor point to retrieve the sensed data since its charging radius is less than the communication radius. However, compared to energy replenishment-only schemes, these joint schemes require additional time spent at each anchor point for MCs to connect with nearby nodes and acquire data. On the other hand, compared to the data collection-only schemes, the utilization of the charging radius instead of the communication radius for grouping nodes increases the number of anchor points, as more clusters or cells are generated. In these existing joint approaches, the process in which the MCs depart from the base station (BS) to recharge all the nodes and gather the data, and subsequently return to the BS to offload the data and prepare for a new round, is referred to as a charging cycle. Obviously, the data collection delay and the node energy replenishment delay are both equivalent to the duration of the charging cycle. The increase in the number of anchor points and the additional time spent at each anchor point make it take longer for the MCs to collect data and replenish nodes’ energy for the whole network. Consequently, this leads to an extended charging cycle. In other words, these joint algorithms inevitably delay data collection and energy replenishment.

In this work, we investigate the problem of scheduling MCs to jointly achieve energy replenishment and data collection while minimizing both data collection and energy replenishment delays. We propose a novel joint energy replenishment and data collection approach that utilizes two types of MCs in collaboration. As shown in Figure 1c, the proposed scheme designates a subset of network nodes as data storage nodes (DSNs) based on their geographic locations. When selecting the DSNs, it is crucial to ensure that if the distance between any other node in the network and the DSN is within the communication radius, these nodes need to send the sensed data to the nearest DSN through single-hop communication. As a result, the DSNs can store all the sensed data in their buffer. A type of MC known as MCDs (mobile charger and data collectors) are then granted access to these DSNs for the purposes of charging and data collection, while another type of MC, known as MCOs (mobile charger only), are assigned to charge the rest nodes (non-DSNs) in the network. In this way, the network’s data collection delay and the energy replenishment delay are both equal to the duration of the MCD’s charging cycle, while the charging delay of the non-DSNs is equal to the duration of the MCO’s charging cycle. Since DSNs are selected based on the communication radius rather than the charging radius, the number of DSNs in the proposed scheme is significantly lower than the number of anchor points in the existing joint approaches, which are dependent on the MC’s charging radius. Consequently, the MCD’s travel distance will be less than the MC’s travel distance in the existing joint approaches, bringing the data collection delay in the proposed approach closer to that of the data collection-only scheme. Furthermore, the MCDs only need to communicate with the data storage nodes to retrieve data instead of individually communicating with each node. This helps to minimize the time spent at the anchor points. Thus, the charging cycle duration of the MCD is greatly reduced compared to that of the MC in the existing joint approaches. As for the MCOs, their residence time at the charging anchor points is significantly shorter than that of the MCs in the existing joint approaches because they are solely responsible for charging and not data collecting. Additionally, since they only charge a part of nodes (non-DSNs) in the network, their travel distance and overall charging time are reduced, resulting in a shorter charging cycle duration.

More specifically, the main contributions of our work can be summarized as follows:

(1) We investigate the joint optimization of energy replenishment and data collection in WRSNs, aiming to minimize both the delay in charging sensor nodes and the delay in data delivery.
(2) We propose a joint optimization scheme based on two successive clustering processes, the first of which clusters the nodes according to their communication radius to select the DSNs responsible for data staging, and the second of which clusters the rest of the nodes according to the charging radius of the MCs to select the charging locations for these non-DSNs. To facilitate efficient energy replenishment and data collection, we introduce two distinct types of MCs to charge the DSNs and non-DSNs, respectively, where the MCD charging DSNs can also collect the data stored by DSN. Finally, a particle swarm approach is used to optimize the paths taken by both MCs.
(3) To validate the effectiveness of the proposed scheme, extensive simulation experiments are conducted, and the results show that the proposed scheme outperforms the existing schemes. The network data delay of the proposed scheme demonstrates improvements of 26.9%, 77.2%, 83.9%, and 32%, respectively, in comparison to the JERDC, SPSS, CIBA-MDSA, and DGCS schemes. Regarding charging efficiency, the proposed scheme exhibits enhancements of 63.3%, 16.5%, 24.7%, and 29.6% relative to the aforementioned schemes. Moreover, the average remaining energy level of the proposed scheme is superior, showing increases of 7.3%, 0.39%, 0.37%, and 0.04% compared to the JERDC, SPSS, CIBA-MDSA, and DGCS schemes, respectively.

The rest of the paper is organized as follows: Section 2 reviews related work; Section 3 introduces the model and formulates our problem; Section 4 presents the proposed scheme; Section 5 simulates the performance evaluation; Section 6 concludes our work.

## 2. Related Work

In this section, we review the previous studies on hierarchical routing in WSNs, charging schemes in hierarchical routing, and joint energy replenishment and data collection schemes, which have served as inspiration for our work.

### 2.1. Charging Schemes Based on Node Grouping

Numerous studies have investigated charging schemes based on node grouping. However, these schemes focused primarily on MC scheduling and did not take into account the impact of data delivery schemes on the speed of node energy consumption. They failed to integrate data delivery with charging schemes. For example, ref. [15] used the low-energy adaptive clustering hierarchy (LEACH) algorithm to group nodes, and they formulated a multi-objective optimization problem for MCs scheduling under a hierarchical network, taking into account conditions such as the number of sensor nodes within the charging ranges, motion energy consumption of the MCs, remaining energy of the nodes, and the distance between MCs and sensor nodes. Finally, an improved firefly algorithm (IFA) and an accurate optimization scheme based on an improved non-dominated sorting genetic algorithm (INSGA-II) were proposed to determine the optimal MC anchor points. Han et al. [16] proposed an uneven cluster-based mobile charging (UCMC) algorithm, which grouped the nodes unevenly to reduce the number of non-functional nodes in the charging schedule. Ref. [17] used the mean-shift algorithm based on density to divide the network into clusters and group nodes, and then the mother wireless charger vehicle (MWCV) carried multiple sub wireless charger vehicles (SWCVs), starting from the base station, and released them when they reached the anchor point. Finally, the SWCVs charged the nodes in each cluster by using a gradient descent optimization algorithm. Ref. [18] proposed an energy- and location-aware K-means (ELAK-means) clustering algorithm and an intra-cluster communication mode-simple tree-like communication mode (STCM) to group the nodes and balance the energy consumption of the nodes. Finally, an MC scheduling algorithm is proposed to schedule MCs to charge nodes with energy below the threshold, which improves charging efficiency. An improved multi-factor fuzzy C-mean clustering algorithm (MFCM++) was used for node grouping [19]. After that, they proposed a cuckoo search algorithm (CS) to determine the optimal charging position for each cluster. Lastly, a spatio-temporal factor-based cluster selection algorithm (STCS) was developed to determine the charging order of clusters and select the next one to be charged.

### 2.2. Joint Energy Replenishment and Data Collection Scheme

As research on WRSNs progresses, an increasing number of researchers recognize the impact of data delivery schemes on the speed of node energy consumption, and consequently, on charging schemes. Therefore, integrating data delivery with the charging scheme has become a new trend in research on WRSNs. A joint data delivery and charging scheme requires MCs to perform two tasks simultaneously: replenishing energy for sensor nodes and collecting the data stored in them. Han et al. [11] proposed a joint energy replenishment and data collection algorithm for WRSNs, which first uses the k-means algorithm to group nodes based on the MCs’ charging radius and uses two MCs to travel simultaneously, along the anchor point from the BS in opposite directions along the shortest Hamiltonian circuit, to collaboratively perform the energy replenishment and data collection tasks. Ref. [12] proposed a joint data collection and energy charging algorithm. They first proposed a node weight-based algorithm to group nodes by the charging radius of MCs, and then developed two scheduling schemes using heuristic algorithms for scenarios with different delay requirements (i.e., delay-tolerant and delay-aware scenarios) to schedule MCs to charge the nodes as well as collect data. Both Refs. [13,14] used hexagonal cells to group nodes, and then proposed the joint scheduling of the MC and UAV for energy replenishment and data collection from the nodes. A discrete fireworks algorithm based on population entropy proposed in [20] addresses joint data collection and energy replenishment. It divides the network area into regular hexagonal cells and considers the center of each region as an anchor point for energy replenishment and data collection. The MC periodically traverses the anchor points to charge the nodes and collect the data. Ref. [21] formulated the data gathering problem as a network utility maximization problem, and proposed a distributed data gathering approach (DDGA), which can obtain the optimal data gathering scheme. In [22], the J-RCA algorithm enhances the TOPSISI routing algorithm to balance node energy consumption and proposes a two-step charging scheme, which consists of calculating the shortest Hamilton cycle of original charging nodes and selecting the other nodes, called passer-by nodes, to fully utilize the energy of the MC. Liu et al. [23] addressed the joint data gathering and energy harvesting (JoDGE) problem and proposed an optimal scheduling scheme. This scheme took into account the power allocation, relay selection, and time slot scheduling policies, aiming to optimize the overall performance of the system. Ref. [24] investigated the periodic energy replenishment and data collection with limited mobile devices (PERDCLMD) problem by weighting the importance of sensor nodes. Additionally, a greedy scheduling scheme was proposed for the PERDCLMD problem. Different from traditional charging scheduling policies, where sensor nodes passively wait for the arrival of mobile vehicles, a novel dynamic clustering based mobile-to-cluster (M2C) scheme was proposed in [25]. The scheme estimates the energy consumption of the data storage nodes and actively selects the sensor nodes with residual energy close to the estimate as data storage nodes, which gives the MC more data storage nodes with low residual energy, reducing the travel distance along with higher energy efficiency of charging.

To ensure MCs simultaneously perform the tasks of data collection and energy replenishment, these studies use clustering algorithms or divide the network area into hexagonal cells to group nodes according to the MCs’ charging radius. This guarantees that when MCs move to the center of a cluster or cell, all nodes within the cluster or cells fall within their charging range, allowing MCs to charge all these nodes in one-to-many mode and collect data from these nodes at once. However, as the communication radius far exceeds the charging radius, clustering or celling based on the charging radius results in a greater number of clusters than clustering based on the communication radius. Consequently, this leads to increased travel distance for MCs, resulting in longer charging and data collection delays.

Table 1 shows the comparison of key parameters considered between our proposed algorithm and the existing algorithms.

## 3. Preliminaries

In this section, we introduce the network model and the energy model. The major notations used in the work are listed in Table 2.

### 3.1. Network Model

As shown in Figure 1c, we consider a two-dimensional area comprising a BS, and *N* stationary sensor nodes. The BS is located at the center of the network and is responsible for uploading data and replenishing energy for MCs. Let $S=\{s_{1},s_{2},...,s_{N}\}$ denote the set of sensor nodes. With a buffer space of $BC_{sensor}$ and a communication radius of $R_{trans}$, each sensor node $s_{i}\in S$ is equipped with a rechargeable battery with the energy capacity of $EC_{sensor}$. Each node senses data at a rate of G(bit/s). Based on the geographic distribution of sensor nodes, our study performs two successive clustering operations (see Section 4 for details). In the first clustering, we group the nodes into clusters according to the communication radius of the sensor node and select one node as the DSN in each cluster. As a result, each node in a cluster can transfer the data it sensed to its DSN in a single-hop way. Let $C=\{C_{1},C_{2},...,C_{K_{DSN}}\}$ denote the set of the clusters, where $K_{DSN}$ is the number of clusters. Without causing confusion, we also use $C_{i}$ to present the selected DSN of the cluster $C_{i}$. In addition, the set of non-DSNs in $C_{i}$ is denoted as $nDSN_{i}=\left\{ndsn_{i,1},ndsn_{i,2},...,ndsn_{i,|nDSN_{i}|}\right\}$, where $nDSN_{i,j}$ denotes the $j^{th}$ non-DSN of $C_{i}$ and $|nDSN_{i}|$ represents the number of non-DSNs in $C_{i}$. In clustering, it is necessary to ensure that each non-DSN belongs to only one cluster, so we have $\sum_{C_{i}\in C}\left|nDSN_{i}\right|+K_{DSN}=N$.

After selecting the DSNs, we perform the second clustering for all the member nodes, according to the charging radius of MCs. Let $C^{\prime }=\left\{C_{1}^{\prime },C_{2}^{\prime },...,C_{K_{nDSN}}^{\prime }\right\}$ denote the set of the clusters obtained from the second clustering, where $K_{nDSN}$ is the number of the clusters. Moreover, $nDSN_{i}^{\prime }=\left\{ndsn_{i,1}^{\prime },ndsn_{i,2}^{\prime },...,ndsn_{i,|nDSN_{i}^{\prime }|}^{\prime }\right\}$ represent the set of non-DSNs in $C_{i}^{\prime }$, where $ndsn_{i,j}^{\prime }$ denotes the $j^{th}$ member nodes of $C_{i}^{\prime }$ and $|nDSN_{i}^{\prime }|$ represents the number of non-DSNs in $C_{i}^{\prime }$. Combining the above, all non-DSNs in the network can be represented as $nDSN={⋃}_{C_{i}\in C}nDSN_{i}={⋃}_{C_{i}^{\prime }\in C^{\prime }}nDSN_{i}^{\prime }$. Without causing confusion, we also use $C_{i}^{\prime }$ to present the coordinate of the geographic center of $C_{i}^{\prime }$. After completing the two successive clustering, we use two distinct types of MCs, MCD and MCO, to charge DSNs and non-DSNs, respectively, and the scheduling cycles of MCD and MCO are denoted as $T_{DSN}$ and $T_{nDSN}$, respectively. Both MCD and MCO utilize rechargeable batteries with the energy capacity of $EC_{mc}$ and MCD is also equipped with a buffer space of $BC_{mc}$ to collect data from the DSN’ cache.

### 3.2. Energy Model

To simplify the expression, we let function d(x,y) denote the Euclidean distance between two entities *x* and *y* (e.g., MC and node, node and node, etc.).

We employ the first-order radio model [26] to model energy consumption for data transmission and reception:

(1) $$E_{tx}\left(l,d(t,r)\right)=\left\{\begin{array}{c} l\times \left(E_{elec}+\varepsilon_{fs}\times d{\left(t,r\right)}^{2}\right),d\left(t,r\right)<d_{0}\\ l\times \left(E_{elec}+\varepsilon_{mp}\times d{\left(t,r\right)}^{4}\right),d\left(t,r\right)\geq d_{0} \end{array}\right.,$$

(2) $$E_{rx}\left(l\right)=l\times E_{elec},$$

where *l* is the size of the transmitted and received data. $E_{tx}$ and $E_{rx}$ denote the energy cost of data transmission and reception, respectively. $E_{elec}$ is the energy consumed to run the transmitter or receiver. According to the distance between the transmitter and the receiver d(t,r), the energy dissipation by the amplifier can be modeled as a free-space or a multipath fading model. $\varepsilon_{fs}$ and $\varepsilon_{mp}$ are used for the free space and multipath fading model, respectively, whereby the free-space model is employed in cases when d(t,r) is less than the threshold $d_{0}$, and the multipath fading model is applied when d(t,r) exceeds the threshold $d_{0}$.

The total energy consumption of MCD comprises three components: motion, charging, and data reception. Thus, the total energy consumption of MCD can be expressed as follows:

(3) $$E_{MCD}^{con}=P_{move}\times \frac{D_{C}}{V_{mc}}+P_{charge}\times \sum_{C_{i}\in C}CT_{C_{i}}+E_{rx}\left(\sum_{C_{i}\in C}\times Q\right),$$

where $P_{move}$ is the traveling power of MC and $P_{charge}$ is the charging power of MC. $D_{c}$ represents the total distance traveled by the MCD in the current cycle. $V_{mc}$ represents the travel speed of the MC. $CT_{C_{i}}$ denotes the duration of MCD charging at the DSN in $C_{i}$. $MT_{C_{i}}$ denotes the duration for which the MC collects cached data from the DSN in $C_{i}$. *Q* is the data collection rate of the MC.

Similarly, the total energy consumption of MCO is as follows:

(4) $$E_{MCO}^{con}=P_{move}\times \frac{D_{C}^{\prime }}{V_{mc}}+P_{charge}\times \sum_{C_{i}^{\prime }\in C^{\prime }}CT_{C_{i}^{\prime }}.$$

The energy consumption of a node is comprised of four main components: data sensing, computing, data transmission, and data reception. The energy consumption for data sensing and computing is negligible compared to that for data transmission and reception. DSNs continuously receive and save the data transmitted by non-DSNs and then transfer the stored data to MCs upon MCs’ arrival. Therefore, the total energy consumption of the DSN in $C_{i}$ is as follows:

(5) $$E_{C_{i}}^{con}=E_{tx}\left(MT_{C_{i}}\times Q,d\left(mc,C_{i}\right)+E_{rx}\left(|nDSN_{i}|\times G\times T_{DSN}\right)\right).$$

Non-DSNs, on the other hand, are only responsible for sensing the data and sending it to the DSN. Thus, the total energy consumption of $ndsn_{i,j}$ is as follows:

(6) $$E_{ndsn_{i,j}}^{con}=E_{tx}\left(T_{nDSN}\times G,d\left(C_{i},ndsn_{i,j}\right)\right).$$

In this paper, we use the charging model from [27]:

(7) $$P_{rev}\left(d(mc,node)\right)=\left\{\begin{array}{c} \frac{P_{charge}\times \alpha }{{\left(d(mc,node)+\beta \right)}^{2}},d\left(mc,node\right)\leq R_{charge}\\ 0,d\left(mc,node\right)>R_{charge} \end{array}\right.,$$

where $P_{rev}$ refers to the received power by node. α and β are constants determined by the charger hardware and environment. $R_{charge}$ is the charging radius of the MC. This charging model is applicable to both one-to-one and one-to-many charging scenarios, as demonstrated in [28]. Therefore, the power received by $ch_{i}$ and $ndsn_{i,j}$ are the following, respectively:

(8) $$P_{C_{i}}=P_{rev}\left(d\left(C_{i},MCD\right)\right),$$

(9) $$P_{ndsn_{i,j}}=P_{rev}\left(d\left(ndsn_{i,j},MCO\right)\right).$$

Since the MCD charges the DSN in a one-to-one pattern, the MCD stays close to the DSN to charge it, i.e., $d\left(C_{i},MCD\right)=0$.

### 3.3. Problem Definition

The goal of our work is to optimize the data delivery delay and the node charging delay by scheduling MCs. The first step in scheduling MCs is to calculate the anchor points where MCs stop to collect data and charge nodes. Then, MCs can plan their path based on these anchor points. As a result, anchor points determination and MC path planning can be seen as two subproblems of the objective.

#### 3.3.1. Anchor Points Determination

We aim to decrease the number of anchor points on MCs’ path in our work to reduce data transmission delay and charging delay, as described earlier. Two successive clustering operations are carried out: one according to the nodes’ communication radius and another according to the MCs’ charging radius. The first clustering determines which nodes will be most effective as the DSNs for temporarily storing the data collected. The MCD then uses these nodes’ locations as anchor points to collect data and charge them. The second clustering determines the anchor points where the MCO stops to charge the non-DSNs. The DSNs and the anchor points for non-DSNs are two kinds of anchor points in our scheme.

a. Determining the DSNs

The number of clusters determines the number of DSNs. A smaller number of DSNs leads to a shorter travel distance for the MCD and a reduced workload. Therefore, our optimization aims for DSNs determining is to minimize the number of clusters. We model the DSNs determining problem as follows:

(10) $$\min K_{DSN},$$

(11) $$s.t.s_{j}\in C_{i},\forall s_{j}\in S,$$

(12) $$\underset{C_{i}\in C}{⋂}C_{i}=\emptyset ,$$

(13) $$d\left(ndsn_{i,j},C_{i}\right)\leq R_{trans},\forall ndsn_{i,j}\in nDSN_{i},\forall C_{i}\in C,$$

where (10) indicates that our optimization objective is to minimize the number of anchors for the MCD. (11) states that each sensor node needs to be assigned to a cluster. (12) means that clusters cannot be empty. (13) guarantees that the nodes in the cluster can communicate with the DSN.

b. Determining anchor points for the MCO

Similar to the DSNs determining problem, fewer clusters obtained from the second clustering can reduce the number of the MCO’s anchor points, which in turn reduces the distance traveled by the MCO. This reduces charging delays and increases the energy levels of non-DSNs. Therefore, the optimization objective for determining anchor points for the MCO is also to minimize the number of clusters. The problem is formulated as follows:

(14) $$\min K_{nDSN},$$

(15) $$s.t.\;ndsn_{i,j}^{\prime }\in nDSN_{k}^{\prime },\forall ndsn_{i,j}^{\prime }\in nDSN,$$

(16) $$\underset{C_{i}^{\prime }\in C^{\prime }}{⋂}C_{i}^{\prime }=\emptyset ,$$

(17) $$d\left(ndsn_{i,j}^{\prime },C_{i}^{\prime }\right)\leq R_{charge},\forall ndsn_{i,j}^{\prime }\in nDSN_{i}^{\prime },\forall C_{i}^{\prime }\in C^{\prime },$$

where (14) indicates that our optimization objective is to minimize the number of anchors for the MCO. (15) states that each non-DSN needs to be assigned to a cluster. (16) means that clusters cannot be empty. (17) guarantees that the size of the non-DSN’s cluster cannot exceed the charging radius.

#### 3.3.2. Scheduling of MCs

When MCs serve nodes, they always start from the BS and end back at the BS. Considering the energy and buffer limitations, MCs travel to the BS for energy replenishment or data offloading due to energy shortage and data overflow, and they then travel to the remaining nodes. Therefore, the whole path of an MC can be divided into several sub-routes, each of which starts and ends at the BS. Let $SP_{C}$ denote the set of all sub-routes of the MCD and $SP_{C^{\prime }}$ denote the set of all sub-routes of the MCO. Each sub-route, denoted as $sp_{C}^{k}$ or $sp_{C^{\prime }}^{k}$, is an ordered sequence of nodes indicating the order in which the MC visits them.

The total time, total energy consumption, and total collected data of the MCD to move along the $k^{th}$ sub-route $sp_{C}^{k}$ and serve the nodes are as follows, respectively:

(18) $$time_{C}^{k}=\frac{D_{C}^{k}}{V_{mc}}+\sum_{C_{i}\in sp_{C}^{k}}CT_{C_{i}}+\sum_{C_{i}\in sp_{C}^{k}}MT_{C_{i}},$$

(19) $$cost_{C}^{k}=P_{move}\times \frac{D_{C}^{k}}{V_{mc}}+P_{charge}\times \sum_{C_{i}\in sp_{C}^{k}}CT_{C_{i}}+E_{rx}\left(\sum_{C_{i}\in sp_{C}^{k}}MT_{C_{i}}\times Q\right),$$

(20) $$buffer_{C}^{k}=Q\times \sum_{C_{i}\in sp_{C}^{k}}MT_{C_{i}},$$

where $D_{C}^{k}$ denotes the total distance traveled by the MCD in $sp_{C}^{k}$.

Similarly, the total time and total energy consumption of the MCO along the $k^{th}$ sub-route $sp_{C^{\prime }}^{k}$ are as follows, respectively:

(21) $$time_{C^{\prime }}^{k}=\frac{D_{C^{\prime }}^{k}}{V_{mc}}+\sum_{C_{i}^{\prime }\in sp_{C^{\prime }}^{k}}CT_{C_{i}^{\prime }},$$

(22) $$cost_{C^{\prime }}^{k}=P_{move}\times \frac{D_{C^{\prime }}^{k}}{V_{mc}}+P_{charge}\times \sum_{C_{i}^{\prime }\in sp_{C^{\prime }}^{k}}CT_{C_{i}^{\prime }}.$$

Based on the time spent on each sub-route, we can calculate the total time of the current cycle of the MCD and MCO as follows:

(23) $$T_{DSN}=\sum_{sp_{C}^{k}\in SP_{C}}time_{C}^{k},$$

(24) $$T_{nDSN}=\sum_{sp_{C^{\prime }}^{k}\in SP_{C^{\prime }}}time_{C^{\prime }}^{k}.$$

When the MC reaches a node and starts charging it, the energy consumed by DSNs and non-DSNs is calculated as follows:

(25) $$E_{C_{i}}^{arrive}=E_{rx}\left(t_{C_{i}}^{charge}\times G\times \left|nDSN_{i}\right|\right),$$

(26) $$E_{ndsn_{i,j}^{\prime }}^{arrive}=E_{tx}\left(t_{ndsn_{i,j}^{\prime }}^{charge}\times G\right),$$

where $t_{C_{i}}^{charge}$ denotes the time when the MCD begins to charge the DSN in $C_{i}$ and $t_{C_{i}^{\prime }}^{charge}$ denotes the time when the MCO begins to charge.

When the MCD reaches a DSN and begins collecting cached data, the amount of data collected by the DSN is as follows:

(27) $$B_{C_{i}}^{arrive}=t_{C_{i}}^{data}\times \left(|nDSN_{i}|+1\right)\times G,$$

where $t_{C_{i}}^{data}$ denotes the time when the MCD begins to receive data from the DSN in $C_{i}$.

The amount of data collected for the current cycle is as follows:

(28) $$B_{C_{i}}^{cache}=T_{DSN}\times \left(|nDSN_{i}|+1\right)\times G.$$

In addition, two decision variables $y_{i,j,k}$ and $z_{i,j,k}$ are defined below:

(29) $$y_{i,j,k}=\left\{\begin{array}{c} 1,\mathrm{if}\;\mathrm{MCD}\;\mathrm{travels}\;\mathrm{from}\;C_{i}\;\mathrm{to}\;C_{j}\;\mathrm{in}\;sp_{C}^{k}\\ 0,else \end{array}\right.,$$

(30) $$z_{i,j,k}=\left\{\begin{array}{c} 1,\mathrm{if}\;\mathrm{MCO}\;\mathrm{travel}\;\mathrm{from}\;C_{i}^{\prime }\;\mathrm{to}\;C_{j}^{\prime }\;\mathrm{in}\;sp_{C^{\prime }}^{k}\\ 0,else \end{array}\right..$$

Notably, $y_{0,i,k}$ and $y_{i,0,k}$ denote the existence of a path from the BS to the DSN in $C_{i}$ and a path from the DSN in $C_{i}$ to the BS in $sp_{C}^{k}$, respectively, and *z* does the same.

Since we used two types of MCs to serve DSNs and non-DSNs, respectively, the scheduling of MCs is in two parts, the scheduling of the MCD and the scheduling of the MCO, respectively.

a. Scheduling of MCD

Our primary objective is to determine the optimal combination of charging sequences, charging durations, and data collection times to minimize the data delay (cycle $T_{DSN}$) of the MCD. Minimizing data delay is crucial in ensuring prompt delivery of collected data, thereby enabling real-time or near-real-time monitoring and analysis:

(31) $$\min T_{DSN},$$

(32) $$s.t.\forall_{C_{i}\in C}E_{C_{i}}^{res}-E_{C_{i}}^{arrive}>0,$$

(33) $$\forall_{C_{i}\in C}B_{C_{i}}^{res}+B_{C_{i}}^{arrive}<BC_{sensor},$$

(34) $$\forall_{C_{i}\in C}E_{C_{i}}^{res}+E_{C_{i}}^{con}+P_{C_{i}}>0,$$

(35) $$\forall_{C_{i}\in C}B_{C_{i}}^{res}+B_{C_{i}}^{cache}-MT_{C_{i}}\leq BC_{sensor},$$

(36) $$\forall_{sp_{C}^{k}\in SP_{C}}cost_{k}^{C}\leq EC_{mc},$$

(37) $$\forall_{sp_{C}^{k}\in SP_{C}}buffer_{k}^{C}\leq BC_{mc},$$

(38) $$\sum_{sp_{C}^{k}\in SP_{C}}\sum_{C_{i}\in C}y_{i,j,k}=\sum_{sp_{C}^{k}\in SP_{C}}\sum_{C_{i}\in C}y_{j,i,k}=1,$$

(39) $$\forall_{sp_{C}^{k}\in SP_{C}}\sum_{C_{i}\in C}y_{0,i,k}=\sum_{C_{i}\in C}y_{i,0,k}=1,$$

where (31) indicates that the optimization objective is to minimize the delay of DSNs. (32) and (33) ensure that when the MCD reaches the DSN, it has sufficient energy to sustain its operations, and that the cached data does not exceed the storage capacity, where $E_{C_{i}}^{res}$ denotes the remaining energy of the DSN in $C_{i}$ and $B_{C_{i}}^{res}$ denotes the cached data of the DSN in $C_{i}$. (34) and (35) ensure that each DSN survives at the end of the cycle and that the cached data does not exceed storage limitations. (36) and (37) are both constraints for the MCD, and they guarantee that the MCD has sufficient energy and cache space in each sub-route, respectively. (38) ensures that each DSN is served by the MCD and only once, i.e., the outgoing and incoming degrees of the DSN are the same and equal to one. (39) states that for any sub-route within the network, the MCD initiates its journey from the BS and concludes it by returning to the BS.

b. Scheduling of MCO

For the scheduling of MCO, we aim to maximize the total remaining energy of non-DSNs at the end of each cycle. This objective implies that we are seeking an efficient allocation of resources to ensure the non-DSNs are charged in a manner that maximizes their overall energy reserves:

(40) $$\min T_{nDSN},$$

(41) $$s.t.\forall_{C_{i}^{\prime }\in C^{\prime },ndsn_{i,j}^{\prime }\in C_{i}^{\prime }}E_{ndsn_{i,j}^{\prime }}^{res}-E_{ndsn_{i,j}^{\prime }}^{arrive}>0,$$

(42) $$\forall_{C_{i}^{\prime }\in C^{\prime },ndsn_{i,j}^{\prime }\in C_{i}^{\prime }}E_{ndsn_{i,j}^{\prime }}^{res}-E_{ndsn_{i,j}^{\prime }}^{con}+\left(CT_{ndsn_{i,j}^{\prime }}\times P_{ndsn_{i,j}^{\prime }}\right)>0,$$

(43) $$\forall_{sp_{C^{\prime }}^{k}\in SP_{C^{\prime }}}cost_{k}^{C^{\prime }}\geq EC_{mc},$$

(44) $$\sum_{sp_{C^{\prime }}^{k}\in SP_{C^{\prime }}}\sum_{C_{i}^{\prime }\in C^{\prime }}z_{i,j,k}=\sum_{sp_{C^{\prime }}^{k}\in SP_{C^{\prime }}}\sum_{C_{i}^{\prime }\in C^{\prime }}z_{j,i,k},$$

(45) $$\forall_{sp_{C^{\prime }}^{k}\in SP_{C^{\prime }}}\sum_{C_{i}^{\prime }\in C^{\prime }}z_{0,i,k}=\sum_{C_{i}^{\prime }\in C^{\prime }}z_{i,0,k}=1,$$

where (40) denotes that our optimization objective is to minimize the delay of non-DSNs. (41) ensures that each non-DSN survives before being served by the MCO. (42) ensures that each non-DSN must survive at the end of the cycle. (43) ensures that MCO has sufficient energy in each sub-route. (44) ensures that each cluster is served by the MCO and only once. (45) states that the MCO must start from BS and return to BS for any sub-route.

## 4. Proposed Algorithm

In this section, we describe our proposed joint optimization scheme for energy replenishment and data collection, which first determines the anchor points of the MCD and MCO through two consecutive clustering processes, respectively, and then performs path planning for the MCD and MCO, respectively, based on the anchor points.

### 4.1. Anchor Points Determination

#### 4.1.1. Determining the DSNs

In this paper, we utilize the mean-shift algorithm [17] to partition the nodes into different clusters and select DSNs. The adoption of the mean-shift algorithm facilitates the division of areas with a higher density of node distribution into individual clusters. This approach is advantageous for enhancing the efficiency of charging and data collection in the MCD process. It allows for optimal coverage of the sensor network area while reducing the number of clusters, consequently decreasing both charging and data collection delays in the MC system. The mean-shift algorithm does not require a pre-defined number of clusters and dynamically adjust the cluster size based on the bandwidth parameter. To promote energy consumption reduction and balance within each network cluster, we designate the node closest to the geometric center of the cluster as the DSN, while the remaining nodes serve as non-DSNs. By gradually reducing the bandwidth of the mean-shift algorithm, we ensure that the distance between any non-DSN within the clusters and its corresponding DSN remains within the communication radius $R_{trans}$. The details of the DSN clustering algorithm are presented in Algorithm 1. After clustering, the MCD uses the DSNs’ locations as anchor points for data collection and charging.

#### 4.1.2. Determining Anchor Points for MCO

Similar to network clustering and DSN selection, we also employ the mean-shift algorithm to recluster the non-DSNs. We ensure that the distance between each non-DSN and the center of the cluster to which it belongs does not exceed the charging radius.

**Figure 2:**
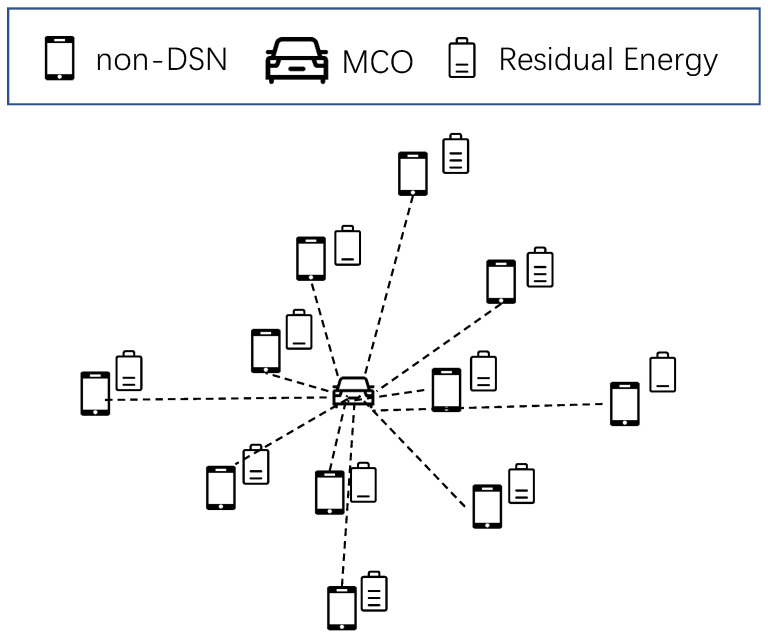
Energy distribution of non-DSNs.

After the second clustering, the non-DSNs covered by MCO may correspond to different DSNs with varying levels of energy consumption and residual energy. An example of the energy distribution of non-DSNs is shown in Figure 2. Additionally, it is important to note that the power received by non-DSNs from MCO decays decreases with distance. To reduce the charging time and maintain energy balance among the nodes following the charging process, it becomes necessary to calculate the anchor point through calculation, which determines the specific positioning of the MCO.
**Algorithm 1:** Algorithms for determining DSN
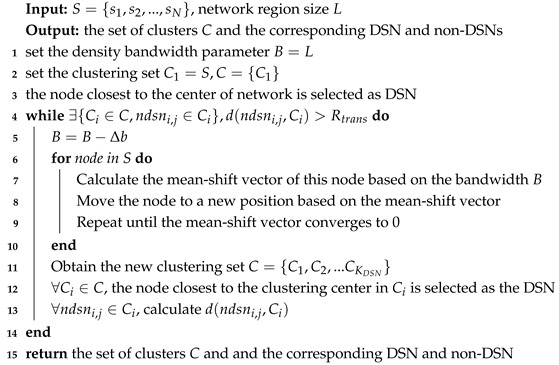


In this paper, non-DSNs with varying residual energies are represented as discrete particles with distinct masses. Nodes with higher residual energy correspond to particles with lower masses. The task of identifying the anchor point for each cluster can be converted into the problem of locating the center of mass within a two-dimensional region.

The coordinates of the anchor point can be obtained through the following procedure:

(46) $$E_{C_{i}^{\prime }}^{total}=\sum_{ndsn_{i,j}^{\prime }\in C_{i}^{\prime }}\left(EC_{sensor}-E_{ndsn_{i,j}^{\prime }}^{res}\right),$$

(47) $$E_{C_{i}^{\prime }}^{y}=\sum_{ndsn_{i,j}^{\prime }\in C_{i}^{\prime }}\left(EC_{sensor}-E_{ndsn_{i,j}^{\prime }}^{res}\right)\times x_{ndsn_{i,j}^{\prime }},$$

(48) $$E_{C_{i}^{\prime }}^{x}=\sum_{ndsn_{i,j}^{\prime }\in C_{i}^{\prime }}\left(EC_{sensor}-E_{ndsn_{i,j}^{\prime }}^{res}\right)\times y_{ndsn_{i,j}^{\prime }},$$

(49) $$\left(x_{C_{i}^{\prime }}^{p},y_{C_{i}^{\prime }}^{p}\right)=\left(\frac{E_{C_{i}^{\prime }}^{y}}{E_{C_{i}^{\prime }}^{total}},\frac{E_{C_{i}^{\prime }}^{x}}{E_{C_{i}^{\prime }}^{total}}\right),$$

where $E_{C_{i}^{\prime }}^{total}$ is the total energy consumed by all non-DSNs in $C_{i}^{\prime }$, $\left(x_{ndsn_{i,j}^{\prime }},y_{ndsn_{i,j}^{\prime }}\right)$ is the coordinates of $ndsn_{i,j}^{\prime }$, $E_{C_{i}^{\prime }}^{y}$ is the sum of static torques of all non-DSNs in the cluster along the y-axis, $E_{C_{i}^{\prime }}^{x}$ is the sum of static torques of all non-DSNs along the x-axis, and $\left(x_{C_{i}^{\prime }}^{p},y_{C_{i}^{\prime }}^{p}\right)$ is the location of the anchoring point where the MCO stops.

When the node distribution is uneven or the network topology is irregular, our proposed mean-shift-based clustering algorithm will tend to divide denser areas into clusters as well as reduce the number of clusters to be divided.

### 4.2. Scheduling for MCs

#### 4.2.1. Scheduling for MCD

This paper presents a periodic scheduling scheme that comprises two distinct parts: the initial cycle and the general cycle.

**Figure 3:**
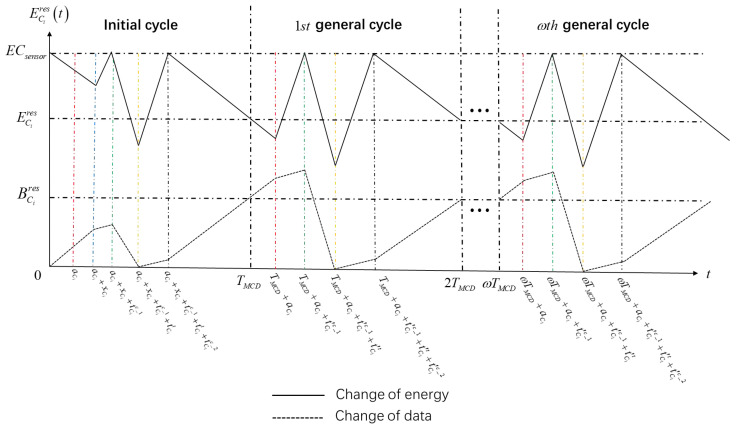
Diagram of energy and data variation of the DSN in $C_{i}$ in periodic charging planning.

In the initial cycle, as depicted in Figure 3, the MCD arrives at the DSN in $C_{i}$ at time $a_{C_{i}}$. To maintain the periodic variation of energy and data within the node, it is necessary for the MCD to undergo a waiting period at the DSN in $C_{i}$ for a duration of $x_{C_{i}}$. Subsequently, the MCD proceeds to replenish energy for the DSN in $C_{i}$ until it reaches full capacity. The charging time, denoted as $t_{C_{i}}^{c\_1}$, is calculated using the following equation:

(50) $$t_{C_{i}}^{c\_1}=\frac{\left(a_{C_{i}}+x_{C_{i}}\right)\times E_{rx}\left(|nDSN_{i}\times G|\right)}{P_{C_{i}}}.$$

Following the first charging phase, the MCD collects the accumulated data stored in the DSN in $C_{i}$. The duration of time, denoted as $t_{C_{i}}^{t}$, required for data collection is calculated through the utilization of the following equation:

(51) $$t_{C_{i}}^{t}=\frac{\left(a_{C_{i}}+x_{C_{i}}\right)\times \left(|nDSN_{i}|+1\right)\times G+t_{C_{i}}^{c\_1}\times G}{Q-G}.$$

To fully charge the DSN in $C_{i}$, the MCD commences another charging process. The duration of the charging time, represented as $t_{C_{i}}^{c\_2}$, can be calculated using the following equation or methodology:

(52) $$t_{C_{i}}^{c\_2}=\frac{t_{C_{i}}^{t}\times E_{tx}\left(Q,d\left(C_{i},MCD\right)\right)}{P_{C_{i}}}.$$

In the general cycle, the arrival time of MCD at the DSN in $C_{i}$ is $\omega T_{DSN}+a_{C_{i}}$. The MCD does not have to wait and directly charges the DSN to full charge. The charging time $t_{C_{i}}^{\prime c\_1}$ is calculated as follows:

(53) $$t_{C_{i}}^{\prime c\_1}=\frac{\left(T_{DSN}-\left(a_{C_{i}}+x_{C_{i}}+t_{C_{i}}^{t}+t_{C_{i}}^{c\_1}+t_{C_{i}}^{c\_2}\right)+a_{C_{i}}\right)\times E_{rx}\left(|nDSN_{i}|\times G\right)}{P_{C_{i}}}.$$

The time $t_{C_{i}}^{\prime t}$ to collect data is calculated as follows:

(54) $$t_{C_{i}}^{\prime t}=\frac{\left(T_{DSN}-\left(a_{C_{i}}+x_{C_{i}}+t_{C_{i}}^{t}+t_{C_{i}}^{c\_1}+t_{C_{i}}^{c\_2}\right)+a_{C_{i}}\right)\times \left(|nDSN_{i}|+1\right)\times G+\left(t_{C_{i}}^{\prime c\_1}+t_{C_{i}}^{c\_2}\right)\times G}{Q-G}.$$

Finally, the time $t_{C_{i}}^{\prime c\_2}$ to fully charge the DSN in $C_{i}$ again is calculated as follows:

(55) $$t_{C_{i}}^{\prime c\_2}=\frac{t_{C_{i}}^{\prime t}\times E_{tx}\left(Q,d\left(C_{i},MCD\right)\right)}{P_{C_{i}}}.$$

To maintain periodic variations in the energy and data of the DSN, it is necessary to ensure that the total time of the initial and general cycles is equal, denoted as $t_{C_{i}}^{c\_1}+t_{C_{i}}^{t}+t_{C_{i}}^{c\_2}+x_{C_{i}}=t_{C_{i}}^{\prime c\_1}+t_{C_{i}}^{\prime t}+t_{C_{i}}^{\prime c\_2}$. In order to achieve this, we can formulate a K-element equation:

(56) $$\begin{array}{cc} t_{C_{1}}^{c\_1}+t_{C_{1}}^{t}+t_{C_{1}}^{c\_2}+x_{C_{1}} & =t_{C_{1}}^{\prime c\_1}+t_{C_{1}}^{\prime t}+t_{C_{1}}^{\prime c\_2}\\ t_{C_{2}}^{c\_1}+t_{C_{2}}^{t}+t_{C_{2}}^{c\_2}+x_{C_{2}} & =t_{C_{2}}^{\prime c\_1}+t_{C_{2}}^{\prime t}+t_{C_{2}}^{\prime c\_2}\\ \; & \vdots \\ t_{C_{i}}^{c\_1}+t_{C_{i}}^{t}+t_{C_{i}}^{c\_2}+x_{C_{i}} & =t_{C_{i}}^{\prime c\_1}+t_{C_{i}}^{\prime t}+t_{C_{i}}^{\prime c\_2}\\ \; & \vdots \\ t_{C_{KDSN}}^{c\_1}+t_{C_{KDSN}}^{t}+t_{C_{KDSN}}^{c\_2}+x_{C_{KDSN}} & =t_{C_{KDSN}}^{\prime c\_1}+t_{C_{KDSN}}^{\prime c}+t_{C_{KDSN}}^{\prime c\_2} \end{array}.$$

This equation is guaranteed to have a unique solution, and the solution $x_{C_{i}}$ represents the waiting time for the initial cycle of the DSN in $C_{i}$.

The scheduling problem of periodic joint energy replenishment and data collection can be effectively addressed through the utilization of a hybrid particle swarm algorithm (HPSA). The HPSA combines the principles of particle swarm optimization (PSO) and genetic algorithms (GAs) to optimize the scheduling process thoroughly.

In order to mitigate the common issue of falling into local optima inherent in PSO, this study introduces a mutation operation inspired by genetic algorithms. By introducing random into the swarm, this operation enables the exploration of alternative solutions beyond the immediate neighborhood, thus enhancing the algorithm’s ability to escape local optima.

Consequently, the proposed hybrid particle swarm optimization algorithm encompasses the following stages:

(1) Initialization: The particle swarm is initialized with randomly generated solutions.
(2) Equations Substitution: Appropriate variable substitutions are made in the Equation (56) to calculate the waiting time, charging time, and data collection time.
(3) Optimal Solution Determination: The optimal solution within the population is determined by evaluating each particle.
(4) Position Update and Mutation: The positions of the particles are updated based on their velocities, and the mutation operation is applied to introduce diversity into the swarm.

The specific algorithm flow is detailed in Algorithm 2.
**Algorithm 2:** scheduling algorithm for MCD
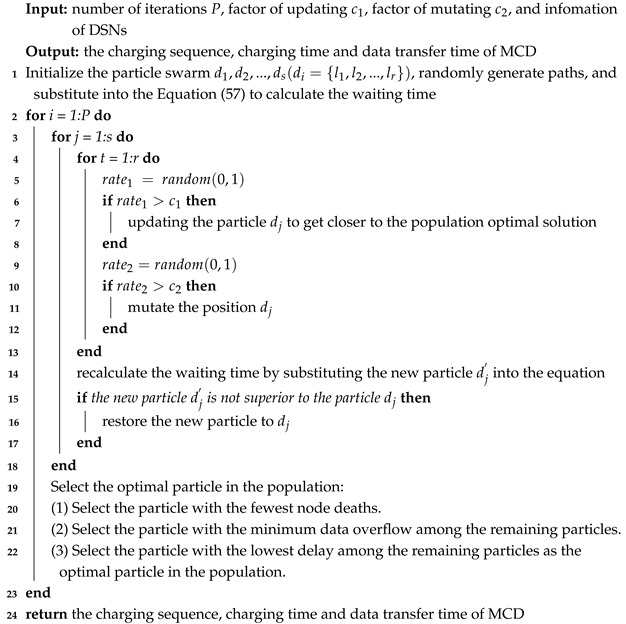


#### 4.2.2. Scheduling of MCO

Since non-DSNs consume much less energy than DSNs, they can tolerate longer charging delays. Moreover, to extend the lifetime of nodes, we adopt a strategy where the nodes in each cluster are fully charged when the MCO reaches the anchor point, which can achieve the long network lifetime [12].

Similar to the scheduling of MCDs, we employ the hybrid particle swarm algorithm to address the scheduling problem of MCOs. We propose the following modifications to the selection process for determining the optimal particle in the population:

(1) Select the particle with the fewest node deaths.
(2) Select the particle with the smallest delay among the remaining particles.

It is important to highlight the distinction between the MCO and the periodic scheduling of the MCD. The scheduling for each cycle is not identical, and at the start of each cycle, it is necessary to reassess anchor points based on the remaining energy of non-DSNs and devise a new path plan.

### 4.3. Time Complexity of the Proposed Algorithm

We use the mean-shift algorithm to determine the DSNs and the anchor points for MCO. As for [29], the time complexity of the mean-shift algorithm is $O(n^{2})$, where *n* is the number of nodes participating in clustering. For determining the DSNs, the number of nodes in clustering is *N*, which is the total number of nodes. Assuming a total of $T_{1}$ iterations are required, the time complexity is $O\left(N^{2}\right)\times T_{1}$. For determining the anchor points for the MCO, the number of nodes in clustering is $N-K_{DSN}$. When calculating anchor points based on energy distribution, it is necessary to calculate the energy of each point. Therefore, the time complexity of this part is $O(N-K_{DSN})$. Assuming a total of $T_{2}$ iterations are required, the time complexity is $O\left({\left(N-K_{DSN}\right)}^{2}\right)\times T_{2}+O\left(N-K_{DSN}\right)$.

The time complexity for scheduling MCDs primarily depends on the number of iterations, the number of particles, and the number of DSNs in the hybrid particle swarm algorithm. Assume that the algorithm iterates $P_{1}$ times and the number of particles is $n_{1}$. When calculating the optimal particle in the population, we need to solve the K-element equation for each particle to calculate the residence time of MCDs, and then compare the current particle with the best particle in the population. When solving the equation, we use the LU decomposition method with a time complexity of $O({\left(K_{DSN}\right)}^{3})$ [30], while the comparison of particle require a time complexity of O(1). Therefore, the time complexity for calculating the optimal particle in the population is $O(n_{1}\times {\left(K_{DSN}\right)}^{3})$. For particle update and mutation operations, as we need to operate on each position of each particle, the time complexity is $O(n_{1}\times K_{DSN})$. The time complexity for other operations of the hybrid particle swarm algorithm is O(1) and can be ignored. Therefore, the time complexity of scheduling MCDs is $\left(O\left(n_{1}\times {\left(K_{DSN}\right)}^{3}\right)+O\left(n_{1}\times K_{DSN}\right)\right)\times P_{1}$. We assume that when scheduling MCOs, the hybrid particle swarm algorithm iterates $P_{2}$ times with a particle count of $n_{2}$, and no equations need to be calculated when scheduling MCOs. Therefore, its time complexity is as follows: $\left(O\left(n_{2}\times K_{nDSN}\right)\right)\times P_{2}$.

## 5. Performance Evaluation

### 5.1. Simulation Environment

In this paper, we evaluate the performance of our proposed scheme by comparing it with the JERDC scheme proposed in [11], the SPSS scheme proposed in [12], the DGCS scheme proposed in [31] and the scheme proposed in [32]. It is worth noting that in [32], we used a combination of the most effective CIBA algorithm and the MDSA algorithm, referred to as the CIBA-MDSA scheme. The JERDC scheme proposed in [11] selects DSNs based on their remaining energy and their distances to their own clustering centers, and deploys two MCs to traverse along the shortest Hamiltonian cycle consisting of all the DSNs in order to collaboratively perform the energy replenishment and data collection tasks. The SPSS scheme proposed in [12] selects the DSNs by considering the sensor’s indegree, distance to neighbors, and residual energy. It uses a genetic algorithm to obtain the path with the highest charging utility, and then divides it into two paths of the same length, traversing both paths simultaneously using two MCs. The CIBA-MDSA scheme [32] uses the location of each node and the intersection of a circle with each node as the center and the charging range as the radius as candidate anchor points. The node with the highest indegree among the candidate anchor points is selected as the anchor point until all nodes are covered. In addition to this, the CIBA-MDSA scheme uses the indegree of the anchor point and the distance from the anchor point to the BS to determine the path for the MC to access the anchor point. The DGCS scheme proposed in [31] first divides the sensor nodes into clusters using the circle covering method and determines the anchor points. The minimum number of MCs is then determined using the elbow method in the k-means algorithm. Finally, a genetic algorithm is used to schedule the MCs for node charging and data collection.

For the selection of MCD and MCO speed, when the speed of the MC rises, its traveling energy consumption rises and the charging delay will show a decreasing trend within a certain range. Whereas the energy of the MC is limited, a rise in its traveling energy consumption will result in a decrease in the proportion of the MC’s capacity used for charging, leading to the MC only being able to charge fewer nodes. Therefore, when the speed of MC continues to increase, the energy consumption of MC for traveling increases, but the charging delay decreases instead. Considering the other side, when the speed of the MC decreases, the traveling power of the MC decreases, but the time taken by the MC to travel the same distance rises. When the power decreases and time rises, the energy consumed by the MC traveling is unknown. Therefore, we chose 1 m/s for our MCD and MCO speeds based on experience as well as references [33,34,35].

Moreover, in the simulation experiments, nodes are deployed in the sensor network by randomization. Therefore, the distribution of nodes may be uniform or non-uniform. Our experimental results demonstrate that our clustering algorithm can effectively handle non-regular topology networks and non-uniformly distributed nodes.

We conducted three sets of simulation experiments to evaluate the performance of our proposed scheme, the JERDC scheme, the SPSS scheme, the CIBA-MDSA scheme, and the DGCS scheme based on the size of the simulation region, the number of nodes and the data sense rate of nodes, respectively, with each set of experiments running for 50 cycles of simulation. Moreover, three criteria are used to present the performance of these algorithms:

(1) Average delay. The network’s data collection delay and the DSN’s charging delay are equal to the duration of the MCD charging cycle, while the charging delay of the non-DSN nodes is equal to the duration of the MCO charging cycle. The average delay is a crucial metric in the joint energy replenishment and data collection optimization scenario [12], which represents the timeliness of network data collection and charging.
(2) Charging efficiency: It is the ratio of the energy used by the MC for charging sensors to the total energy consumed by the MC. This metric measures how efficiently the MC utilizes its energy resources to charge the sensor nodes within the network [36].
(3) Average residual energy of nodes: It is the average residual energy of all nodes at the end of the simulation. A larger average residual energy among all nodes when the simulation is terminated indicates a longer network lifetime. This metric is used to evaluate the performance of the energy efficiency in WRSNs [12].

The parameters used in the simulation are shown in Table 3.

### 5.2. Performance Analysis

#### 5.2.1. Varying the Size of Simulation Region

**Figure 4:**
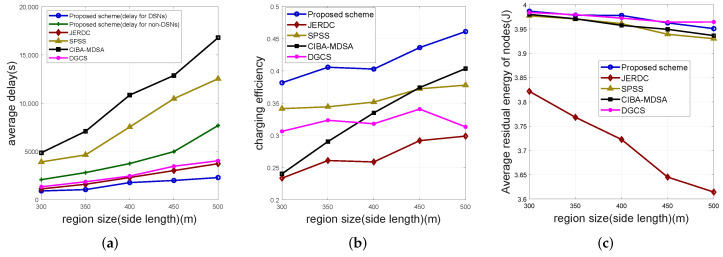
Impact of the size of simulation region. Where (**a**) depicts the simulation results of average delay, (**b**) depicts the simulation results of charging efficiency and (**c**) shows the simulation results of nodes’ average residual energy.

In Figure 4, we set the size of simulation region to 300 m, 350 m, 400 m, 450 m, and 500 m, the number of nodes to 400, and the data sense rate of nodes to 128 bit/s. We set the size of the simulation region as the horizontal coordinates of the figures.

Figure 4a presents a clear trend where the average delay of all schemes experiences an increment as the region size is varied from 300 m to 500 m. This increase in delay can be primarily ascribed to the expansion of the region, which leads to a higher number of anchor points. The increased number of anchor points, in turn, lengthens the path length traversed by the MCs. Significantly, within the proposed scheme, the delay of DSNs attains the lowest value among all the schemes under consideration. This indicates that the network data collection delay of the proposed scheme is also the minimum. The underlying reason for this favorable outcome lies in the utilization of the communication radius instead of the charging radius during the selection of DSNs. This substitution effectively reduces the number of anchor points associated with the MCD, thereby reducing the travel distance of the MCD. Concerning the non-DSNs, their delay is slightly higher than that of the JERDC scheme but remains notably lower than the other two schemes. The relatively low delay of the JERDC scheme can be attributed to its employment of two MCs to partially charge the nodes along the shortest Hamiltonian cycle, which effectively curtails the delay. In contrast, the DGCS scheme deploys a greater number of MCs to serve the nodes, achieving a lower delay as well. Nevertheless, it is crucial to note that the network data collection delays of both the JERDC and DGCS schemes exceed that of the proposed scheme. The SPSS scheme and the CIBA-MDSA scheme exhibit higher delays, primarily because the delay factor was not incorporated into their path planning processes. When evaluating the rate of increase in delay, the delay of DSNs in the proposed scheme grows at a slower pace. This can be explained by the fact that, as the simulation area expands, the number of DSNs in the proposed scheme increases more gradually compared to other schemes. Coupled with its proficient path planning strategy, this results in the most favorable rate of increase in DSN delay. In contrast, the delay of non-DSNs in the proposed scheme grows slightly faster than that of the JERDC and DGCS schemes. This phenomenon can be explained by the fact that the JERDC and DGCS schemes utilize multiple MCs, whereas the non-DSNs in the proposed scheme rely solely on a single MCO for charging. Despite this, the growth rate remains within an acceptable range. In comparison, the SPSS and CIBA-MDSA schemes witness a relatively rapid increase in delay. This is mainly due to the significantly faster growth of the number of anchors in these schemes, compounded by the inferior performance of their path planning algorithms. As the region size continues to expand, it can be reasonably anticipated that the DSN delay of the proposed scheme will maintain its superiority over other schemes. Similarly, the JERDC and DGCS schemes will continue to outperform the non-DSN schemes in terms of delay. Conversely, the performance of the SPSS scheme and the CIBA-MDSA scheme will progressively deteriorate.

Figure 4b shows that as the area size increases, the path length of the MC indeed exhibits an upward trend. However, it is notable that the charging efficiencies of all schemes, except for the DGCS scheme, demonstrate an increasing tendency. This phenomenon can be attributed to the fact that as the network area expands, the waiting time of nodes also increases. Consequently, the MC is required to remain at the charging position for a longer duration, thereby resulting in an increase in the proportion of energy utilized by the MC for charging and ultimately enhancing the charging efficiency. In contrast, the DGCS scheme exhibits a fluctuating charging efficiency. This is primarily due to its utilization of multiple MCs. As the area increases, the increase in the charging time per MC is not substantial, leading to the observed fluctuations in charging efficiency. The proposed scheme exhibits superior charging efficiency compared to several other schemes. This superiority can be attributed to two key factors. Firstly, the proposed scheme features a relatively shorter path length for the MC. Secondly, it employs full charging, which enables the full utilization of the MC’s energy. Among the other schemes, the SPSS scheme and the CIBA-MDSA scheme, although they also adopt full charging, suffer from lower charging efficiencies due to the longer paths traversed by their MCs. The DGCS scheme demonstrates the lowest charging efficiency. This is because it deploys multiple MCs, and each MC’s energy used for charging is relatively low, thus resulting in inefficiency. The JERDC scheme is the least efficient as it employs partial charging, which leads to the underutilization of the MC’s energy. Regarding the rate of increase in charging efficiency, the proposed scheme continues to experience an increase. The SPSS scheme and the JERDC scheme display a relatively slower rate of increase. The DGCS scheme exhibits fluctuations. The CIBA-MDSA scheme initially increases at a rate similar to that of the proposed scheme. However, in experiments conducted in larger regions, its rate of increase in charging efficiency is bound to slow down due to the inferior performance of its path optimization component. In conclusion, as the region size continues to expand, the proposed scheme maintains its superiority in terms of charging efficiency over several other schemes.

Figure 4c illustrates that as the network expands, the residual energy of the nodes tends to decrease. This can be primarily attributed to the elongation of the MC path length, which leads to a longer node charging delay. Consequently, the nodes consume more energy during the simulation process, leading to a reduction in their residual energy at the end. Notably, the nodes in the proposed scheme maintain a consistently high level of residual energy. This advantageous outcome can be ascribed to the independent charging mechanisms designed specifically for DSNs and non-DSNs in our proposed scheme. These mechanisms effectively balance the energy consumption between the two types of nodes, thereby ensuring a relatively stable energy reserve. The SPSS, CIBA-MDSA, and DGCS schemes also exhibit a relatively high level of residual energy. This is mainly due to their use of the full charging approach, which enables the nodes to receive sufficient energy replenishment. In contrast, the JERDC scheme demonstrates the lowest residual energy among all the schemes. The root cause of this lies in its utilization of the partial charging mechanism, which fails to fully meet the energy requirements of the nodes. When considering the rate of decrease in residual energy, the JERDC scheme exhibits the most significant decline. This is an inevitable consequence of its partial charging method. In contrast, the remaining three schemes, namely the proposed scheme, the SPSS scheme, and the CIBA-MDSA scheme, display a similar rate of decrease. This similarity can be explained by their shared use of the full charging approach. In summary, as the area size continues to increase, the proposed scheme, along with the SPSS, CIBA-MDSA, and DGCS schemes, will maintain higher energy levels. In contrast, the residual energy of the JERDC scheme will remain lower compared to the other four schemes.

#### 5.2.2. Varying the Number of Nodes

**Figure 5:**
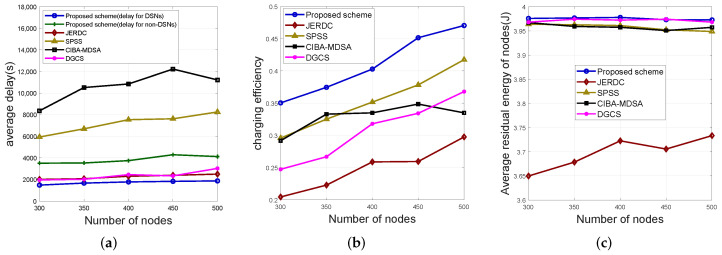
Impact of the number of nodes. Where (**a**) depicts the simulation results of average delay, (**b**) depicts the simulation results of charging efficiency and (**c**) shows the simulation results of nodes’ average residual energy.

In Figure 5, we set the number of nodes to 300, 350, 400, 450, and 500, the size of simulation region to 400 m, the data sense rate of nodes to 128 bit/s, and the number of nodes as the horizontal coordinates of the figures.

Figure 5a clearly indicates that as the number of nodes increases within the range from 300 to 500, a consistent upward trend in delay is observed across all scenarios. This can be primarily attributed to the fact that an increase in the number of nodes leads to a corresponding growth in the data collected by the MC in all scenarios. Concurrently, the energy consumption of the DSNs also rises. These factors jointly result in an inevitable increase in both the charging time and the data reception time, thereby giving rise to the observed increase in delay. In the proposed scheme, it is notable that the delay of the DSNs remains consistently lower than that of the other schemes. This favorable outcome can be ascribed to the utilization of communication radius clustering in the proposed scheme. This clustering method effectively reduces the number of anchors, thus contributing to the delay reduction. Although the delay of the non-DSNs is slightly higher than that of the JERDC and DGCS schemes, it should be noted that both the JERDC and DGCS schemes employ multiple MCs, whereas the proposed scheme utilizes only a single MCO to charge the non-DSNs. Moreover, considering that the DSN delay in the proposed scheme is lower than that of the JERDC and DGCS schemes, this slight increase in non-DSN delay can be regarded as an acceptable trade-off. In contrast, the delays of the SPSS and CIBA-MDSA schemes are significantly higher than those of the other three schemes. This is primarily because these programs fail to take into account the impact of delay during the path planning process. When analyzing the growth rate of delay with respect to the increase in the number of nodes, it is found that the delay of the DSNs in the proposed scheme exhibits the lowest growth rate. Meanwhile, the growth rate of the non-DSNs is slightly higher than that of the JERDC and DGCS schemes. The SPSS scheme continues to experience a relatively high growth rate. For the CIBA-MDSA scheme, although the delay decreases when the number of nodes reaches 500, its inferior path planning algorithm implies that as the number of nodes continues to increase further, the delay is bound to increase. In conclusion, as the number of nodes continues to grow, the DSNs in the proposed scheme will maintain lower delay, and the non-DSN delay will remain lower than in the SPSS and CIBA-MDSA schemes.

Figure 5b reveals that as the number of nodes increases, the charging efficiencies of these schemes generally display an upward trend. This can be primarily ascribed to the augmented energy consumption of the DSNs, which prompts the MC to allocate more energy for node charging. When the number of nodes is subject to variation, the charging efficiency of the proposed scheme consistently outperforms that of several other schemes. This superiority can be attributed to the shorter path length and the full charging mechanism implemented in the proposed scheme. Although the SPSS, CIBA-MDSA, and DGCS schemes also adopt the full charging mechanism, their charging efficiencies remain lower than that of the proposed scheme due to the longer travel distances of the MC. The JERDC scheme exhibits the lowest charging efficiency on account of its partial charging mechanism. Regarding the rate of increase in charging efficiency, the proposed algorithm experiences a more rapid growth as the number of nodes rises. The DGCS, SPSS, and JERDC schemes follow with the next highest rates of increase. In contrast, the CIBA-MDSA scheme demonstrates the lowest rate of increase in charging efficiency, which can be attributed to the suboptimal performance of its path planning algorithm. Consequently, as the number of nodes continues to expand, the proposed algorithm is expected to maintain the highest charging efficiency among these schemes.

Figure 5c demonstrates that a decreasing tendency in node residual energy is exhibited by all schemes, with the exception of the JERDC scheme. The reason for this anomaly in the JERDC scheme lies in its employment of a partial charging mechanism. When the number of nodes increases, the DSNs in the JERDC scheme consume more energy. Concurrently, the MC may spend a longer time in the vicinity of these nodes, and the non-DSNs receive more energy, thereby resulting in an overall increase in node energy. However, as the number of nodes gradually increases, the charging delay of the nodes also rises. This delay can potentially lead to a reduction in the residual energy of the nodes. As a consequence, the average residual energy of the nodes in the JERDC method fluctuates within a specific range. For the remaining schemes, the higher energy consumption of DSNs and the need for MCs to collect more data necessitate that the nodes require more time to replenish their charge. This ultimately leads to a decrease in the average residual energy of the nodes. In contrast, the proposed scheme yields a relatively higher residual energy of the nodes. This is attributable to the utilization of distinct charging mechanisms for DSNs and non-DSNs.When the number of nodes is on the rise, the proposed scheme and the DGCS scheme exhibit the slowest decreasing trends. The SPSS and CIBA-MDSA schemes display a slightly higher decreasing trend. The JERDC scheme, due to its partial charging mechanism, maintains the lowest and most fluctuating level of node energy. In conclusion, as the number of nodes continues to increase, the node residual energy of the proposed algorithm will consistently remain higher than that of several other schemes.

#### 5.2.3. Varying the Amount of Data Sense Rate of Nodes

**Figure 6:**
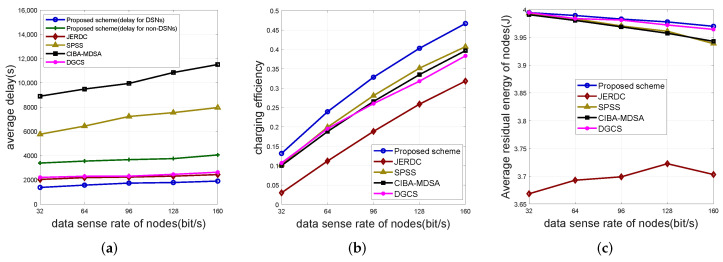
Impact of the number of nodes. Where (**a**) depicts the simulation results of average delay, (**b**) depicts the simulation results of charging efficiency and (**c**) shows the simulation results of nodes’ average residual energy.

In Figure 6, we set the data sense rate of nodes to 32 bit/s, 64 bit/s, 96 bit/s, 128 bit/s, and 160 bit/s, the number of nodes to 400, the size of simulation region to 400 m, and the data sense rate of the nodes as the horizontal coordinates of the figures.

As depicted in Figure 6a, when the data sensing rate of the node is altered within the range from 32 bit/s to 160 bit/s, an increasing tendency in the delay of all scenarios is observed. This phenomenon is mainly due to the elevation of the node’s data sensing rate, which consequentially gives rise to an augmentation in energy consumption and the volume of data cached by the node. Subsequently, the time expended by the MC for charging the nodes and collecting data is prolonged, thereby culminating in an increase in delay. Furthermore, in the proposed scheme, the employment of the minimum number of anchors effectively curtails the path length of the MCDs. This reduction, in turn, mitigates the charging delay for DSNs and the data collection delay of the network. In the JERDC scheme, the strategy of deploying two MCs traveling in opposite directions manifests a marginal reduction in delay when contrasted with the approach of utilizing only a single MCO for charging non-DSNs. The DGCS scheme exhibits lower delay in comparison to non-DSNs due to the utilization of a greater number of MCs. Although the JERDC and DGCS schemes display slightly lower delay relative to non-DSNs, their delay remains higher than that of DSNs. This implies that the data collection delay of the network in these schemes exceeds that of the proposed algorithm. In contrast, the deficiencies in the path planning algorithms of the SPSS and CIBA-MDSA schemes precipitate significantly elevated delays. Regarding the rate of increase in delay, the proposed algorithm, along with the delays of DSNs and non-DSNs in it, as well as those of the JERDC and DGCS schemes, exhibit a relatively stable and gradual increase. In contrast, the SPSS and CIBA-MDSA schemes experience a more pronounced rate of increase in delay. Consequently, the proposed scheme also demonstrates superior performance even when the data sensing rate continues to escalate.

In Figure 6b, as the data sensing rate of the node increases, its energy consumption rises proportionally. Consequently, the MC is required to supply a greater amount of charge to the node. This leads to an upward trend in the charging efficiency of each scheme. The proposed algorithm demonstrates a higher charging efficiency. This is primarily attributed to its utilization of full charging and a shorter path traversed by the MC. Although the SPSS, CIBA-MDSA, and DGCS schemes also adopt full charging, their charging efficiencies are lower than that of the proposed scheme. This is due to the suboptimal performance of their path planning algorithms. In contrast, the JERDC scheme employs partial charging, which results in the lowest charging efficiency among all the schemes. Regarding the rate of increase in charging efficiency, these algorithms exhibit relatively similar trends. Therefore, as the node sensing rate continues to escalate, the proposed algorithm continues to display the highest charging efficiency.

Figure 6c illustrates a decreasing trend in the residual energy of nodes in the proposed SPSS, CIBA-MDSA, and DGCS schemes. This can be primarily attributed to the fact that the energy consumption of the node is directly proportional to the data sensing rate. As the node’s energy consumption increases, the charging time of the MC increases, thereby resulting in a longer charging delay. This delay, in turn, leads to a reduction in the node’s residual energy. In contrast, the JERDC scheme, which employs a partial charging mechanism, experiences a different scenario. In this case, the energy consumption of the DSNs grows at a faster rate than that of the non-DSNs. As a consequence, the MC remains at the anchor point for a longer duration, enabling it to supply more power to the node. This ultimately leads to an increase in the node’s residual energy. However, when the data sensing rate of the node reaches 160 bits/s, the node’s energy consumption becomes excessively high, potentially causing a decrease in the residual energy of the node. It is particularly remarkable that the proposed algorithm exhibits the maximum residual energy among the nodes. This is due to the innovative separate charging mechanisms for DSNs and non-DSNs. In the DGCS scheme, although it utilizes more MCs and full charging, its nodes’ residual energy is slightly lower than that of the proposed algorithm. The SPSS and CIBA-MDSA schemes, despite also employing full charging, possess lower residual energy levels in their nodes compared to the DGCS scheme. This is primarily because of the inferior performance of their path planning methods. On the other hand, the JERDC scheme demonstrates the least residual energy in its nodes due to the use of partial charging. As the node sensing rate continues to increase, the node energy levels in the proposed scheme and the DGCS scheme decreases at a similar rate. In contrast, the node energy levels in the SPSS and CIBA-MDSA schemes decreases at a faster pace. Meanwhile, the node energy level in the JERDC scheme remains relatively low and fluctuates within a certain range due to the partial charging mechanism. In conclusion, the proposed scheme is capable of maintaining a higher level of node residual energy even as the node sensing rate continues to rise.

#### 5.2.4. The Cost of the Schemes

As shown from Table 4, CIBA-MDSA uses the fewest MCs in the simulation environment of this paper, which is only one. This also explains why its network delay is much higher than that of several other schemes. The proposed scheme, the JERDC scheme, and the SPSS scheme use the same number of MCs—two. The proposed scheme has more significant advantages in terms of network delay, charging efficiency, and node residual energy due to the innovative mechanism of using two types of MCs. The DGCS scheme uses the highest number of MCs—three—and thus its performance in terms of network delay and node residual energy is superior. The proposed scheme uses fewer MCs and has a lower cost as compared to DGCS. Moreover, the MCO in the proposed scheme does not need to carry a data collector, making its cost slightly lower than that of the JERDC and SPSS schemes. The CIBA-MDSA scheme has the worst network performance, though it uses only one MC and has the lowest cost. Therefore, the proposed scheme balances the network’s performance and cost.

## 6. Conclusions and Future Work

This paper proposes a joint optimization scheme for energy replenishment and data collection. The scheme first selects DSNs and non-DSNs and then innovatively employs two different MCs (MCD and MCO) to serve these two types of nodes, which reduces the workload and travel distance of the MCs by combining the capabilities of two different MC types. The scheme’s performance demonstrates its potential for optimizing the charging and data transmission strategies, thereby enhancing the overall efficiency of the wireless rechargeable sensor network. In the future, we will focus on the following points for further research:

1. Impacts of real terrain, obstacles, and node failures: in the real world, rolling terrain, obstacles, and the possibility of node failures can have a significant impact on the network. In the future, we will consider these effects in order to further connect the scheme to the real world.
2. Proposed scheme in large-scale networks: In large-scale networks, there is a need to increase the number of MCDs and MCOs to serve the nodes in time and ensure the performance of the network. In larger and more extreme scale networks, there will be node unreachability. We need to optimize the location of the base station so that the MC covers all the nodes in order that the nodes can be served by the MC. In sparse networks, the nodes will be clustered individually and only the MCD is needed in the network without the MCO. How to schedule the MCD for this scenario is another one of our future research directions.
3. Clustering algorithm in the proposed scheme: The clustering algorithm is the core of our proposed scheme; we cluster the nodes based on their geographical location using a density-based approach. In the future, we will consider other factors of the nodes and assign weights to the nodes to improve the performance of the clustering algorithm.
4. Node mobility: We have considered static networks in order to simplify the model. In future work, we will consider dynamic networks where nodes are movable to further optimize the algorithm.

## Figures and Tables

**Table 1:** Comparison of our algorithm and the existing algorithm.

Paper	Multiple Types of MCs ^1^	Multiple Clustering ^2^	Joint Optimization ^3^	Node Grouping ^4^	Anchor Points ^5^
[11]	No	No	Yes	Yes	Yes
[12]	No	No	Yes	Yes	No
[13]	Yes	No	Yes	Yes	No
[14]	Yes	No	Yes	Yes	No
[15]	No	No	No	Yes	Yes
[16]	No	No	No	Yes	No
[17]	Yes	No	No	Yes	Yes
[18]	No	No	No	Yes	No
[19]	No	No	No	Yes	Yes
[20]	No	No	Yes	No	Yes
[21]	No	No	Yes	No	No
[22]	No	No	Yes	No	No
[23]	No	No	Yes	No	No
[24]	No	No	Yes	No	No
[25]	No	No	Yes	Yes	No
Ours	Yes	Yes	Yes	Yes	Yes

^1^ “Multiple types of MCs” indicates whether multiple MCs of different types are used to serve the node. ^2^ “Multiple clustering” indicates whether the network is clustered multiple times. ^3^ “Joint optimization” indicates whether the data delivery scheme is jointly optimized with the charging scheme. ^4^ “Node grouping” indicates whether to group nodes first before scheduling MCs. ^5^ “Anchor points” indicates whether the anchor points for the MCs’ stay are calculated.

**Table 2:** List of notations.

Notation	Description
*S*	The set of nodes
$EC_{sensor}$	Battery capacity of nodes
$BC_{sensor}$	Buffer capacity of nodes
$EC_{mc}$	Battery capacity of MCs
$BC_{mc}$	Buffer capacity of MCs
$R_{trans}$	Communication radius of nodes
$R_{charge}$	Charging radius of MCs
$T_{DSN}$	Scheduling cycles of MCD
$T_{nDSN}$	Scheduling cycles of MCO
*N*	The number of nodes
*G*	Data sense rate of nodes
*Q*	Data collection rate of MCD
$d_{0}$	Threshold of communication distance
$K_{DSN}$	The number of first clusters
$K_{nDSN}$	The number of second clusters
*C*	The set of the clusters obtained from the first clustering
$C^{\prime }$	The set of the clusters obtained from the second clustering
$nDSN_{i}$	The set of non-DSNs in $C_{i}$
$nDSN_{i}^{\prime }$	The set of non-DSNs in $C_{i}^{\prime }$
nDSN	The set of all non-DSNs
$ndsn_{i,j}$	The $i^{th}$ non-DSN in $C_{i}$
$ndsn_{i,j}^{\prime }$	The $i^{th}$ non-DSN in $C_{i}^{\prime }$
$E_{tx}$	Energy spent on data transmission
$E_{rx}$	Energy spent on data reception
$E_{C_{i}}^{con}$	Total energy consumption of the DSN in $C_{i}$
$E_{ndsn_{i,j}}^{con}$	Total energy consumption of $ndsn_{i,j}$
$CT_{C_{i}}$	Duration of MCD charging at the DSN in $C_{i}$
$CT_{ndsn_{i,j}}$	Duration of MCO charging at $ndsn_{i,j}$
$MT_{C_{i}}$	Duration of MCD receiving data at the DSN in $C_{i}$
$P_{charge}$	Energy transfer power of MCs
$P_{move}$	Traveling power of MCs
$D_{C}$	Distance traveled by MCD
$D_{C^{\prime }}$	Distance traveled by MCO
$V_{mc}$	Speed of MCs
$P_{C_{j}}$	Power received by the DSN in $C_{j}$
$P_{ndsn_{i,j}}$	Power received by $ndsn_{i,j}$

**Table 3:** Simulation parameters.

Parameter	Value
$E_{elec}$	50 × 10^−9^ J/bit [26]
$\epsilon_{mp}$	0.0013 × 10^−12^ J/bit/m^4^ [26]
$\epsilon_{fs}$	1 × 10^−11^ J/bit/m^2^ [26]
$E_{sense}$	45 × 10^−9^ J/bit/ [16]
*G*	[32 bit/s, 64 bit/s, 96 bit/s, 128 bit/s, 160 bit/s]
α	0.64 [27]
β	30 [27]
$P_{charge}$	50 W [20]
$V_{mc}$	1 m/s [33,34,35]
$P_{move}$	10 W
$R_{trans}$	120 m [37]
$R_{charge}$	40 m [12]
$d_{0}$	92 m [37]
*L*	[300 m, 350 m, 400 m, 450 m, 500 m]
*N*	[300, 350, 400, 450, 500]
$E_{mc}$	18 KJ
$E_{sensor}$	4 J
*Q*	100 KB/s
$B_{mc}$	10 MB
$B_{sensor}$	2 MB

**Table 4:** The number of MCs used in different schemes.

Schemes	No. of MCs in Var. Size	No. of MCs in Var. Nodes	No. of MCs in Var. Rate
Proposed scheme	2	2	2
JERDC	2	2	2
SPSS	2	2	2
CIBA-MDSA	1	1	1
DGCS	3	3	3

## Data Availability

Data are contained within the article.

## Abbreviations

The following abbreviations are used in this manuscript:

MC	Mobile charger
MCD	Mobile charger and data collectors
MCO	Mobile charger only
DSN	Data storage node
WRSN	Wireless rechargeable sensor network
BS	Base station
